# Association between the expression of specific microRNAs and prostate cancer progression- a systematic review and meta-analysis

**DOI:** 10.3389/fonc.2024.1481885

**Published:** 2025-02-07

**Authors:** Jihad Awadallah Alrehaili

**Affiliations:** Department of Pathology, College of Medicine, Imam Mohammad Ibn Saud Islamic University (IMSIU), Riyadh, Saudi Arabia

**Keywords:** microRNA, prostate cancer, metastasis, gene polymorphism, biomarker

## Abstract

**Background:**

This systematic review and metanalysis aimed to summarize the evidence supporting the significance of particular MiRNAs in PrCa progression and to thoroughly examine the body of prior research.

**Methods:**

In accordance with the PRISMA guidelines, this review was conducted using a specifically design data extraction protocol and searching several online databases for relevant articles. The protocol was registered in the PROSPERO database (CRD42023428460).

**Results:**

8 studies were ultimately included in this review. MiRNAs significantly reduced PrCa proliferation, with an odds ratio (OR) of 0.28 (95% CI: 0.21–0.39) and a risk ratio (RR) of 0.51 (95% CI: 0.43–0.61), though moderate heterogeneity was observed (I² = 57%). For two studies investigating MiRNAs as biomarkers for predicting metastasis, the pooled OR was 0.60 (95% CI: 0.47–0.76) and the RR was 0.72 (95% CI: 0.62–0.84), both demonstrating significant predictive value with low heterogeneity (I² = 0%).

**Conclusion:**

The results emphasize the potential of miRNAs as biomarkers for predicting PrCa metastasis and demonstrate that miRNAs have a discernible effect on PrCa proliferation. However, to improve our comprehension of MiRNA’s function in this condition, additional studies are required to address the limitations and investigate how MiRNA acts in many areas of PrCa.

**Systematic review registration:**

https://www.crd.york.ac.uk/prospero/, identifier CRD42023428460.

## Introduction

Prostate cancer (PrCa) is a prevalent malignancy among men worldwide, and understanding its statistics is crucial for assessing its impact on public health (1). In 2020, there were an estimated 1.4 million new cases of PrCa diagnosed globally (2). It primarily affects older men, with the majority of cases diagnosed in individuals over the age of 65 (2). However, it is relatively rare in men under the age of 40. PrCa is a significant cause of cancer-related deaths in men, accounting for nearly 4% of all cancer-related deaths in 2020 (2). Several risk factors influence the development of PrCa, including age, family history, genetic mutations, and ethnicity, with African American men having a higher incidence and mortality rate (2). Screening for PrCa often involves prostate-specific antigen (PSA) testing, although its effectiveness and potential harms are still a subject of debate (3). Treatment options for prostate cancer depend on various factors, including disease stage, patient age, overall health, and personal preferences (4).

MicroRNAs (MiRNAs) are small non-coding RNA molecules that functionally regulate gene expression through degradation or translational repression of mRNA targets (5). These molecules play important regulatory functions during cellular processes, including cell cycle entry and progression, differentiation, and apoptosis (6). The dysregulation of miRNAs has been causatively involved with tumor initiation, aggressiveness, and therapeutic evasion observed in cancer, particularly with prostate cancer (PrCa). They control proliferation and regression of a tumor with metastasis and drug sensitivity mainly through modulating complex signaling pathways and networks controlling gene expression (7, 8).

MiRNAs in PrCa exert oncogenic and tumor-suppressive functions (9). For instance, some of them are overexpressed, thereby enhancing tumor proliferation and survival, while others are downregulated with lost tumor-suppressive functions (10). Further evidence for the critical roles they play in prostate tissue comes from tissue-specific expression patterns, since aberrant miRNA profiles have been associated with disease progression, aggressiveness, and prognosis in prostate cancer (7, 9). Modified MiRNA expression patterns shed light on their potential use as a diagnostic biomarker, therapeutic target, and prognostic indicator in PrCa (10).

Despite all such developments, translation of MiRNA studies to the clinic remains a challenge. Many studies have small sample sizes, non-standardized methodologies, and heterogeneity in MiRNA selection and analysis, which makes it difficult to compare studies and have robust meta-analyses (14, 15). Most of the studies conducted focus on single MiRNAs without taking into account their interactions in complex regulatory networks, thereby limiting understanding of their roles in broader PrCa (3, 9). Sample collection, RNA extraction, and detection platforms also vary across studies (10).

Variations in study designs, including sample collection methods, RNA extraction techniques, and MiRNA detection platforms, contribute to inconsistencies and make comparisons challenging. Addressing these literature gaps through well-designed studies with larger sample sizes, standardized methodologies, and prospective designs would enhance the reliability and generalizability of findings. Therefore, the objectives of this investigation were to comprehensively evaluate the existing literature and summarize the evidence regarding the role of specific MiRNAs in PrCa progression. These studies aimed to identify and synthesize relevant scientific research to investigate the association between specific MiRNAs and PrCa progression, providing an overview of the current knowledge in the field. Additionally, the investigation secondarily aimed to explore potential sources of heterogeneity across studies, such as study design, sample size, methodology, and patient characteristics, and assess the quality and reliability of the included studies.

## Materials and methods

### Review protocol

**Figure 1:**
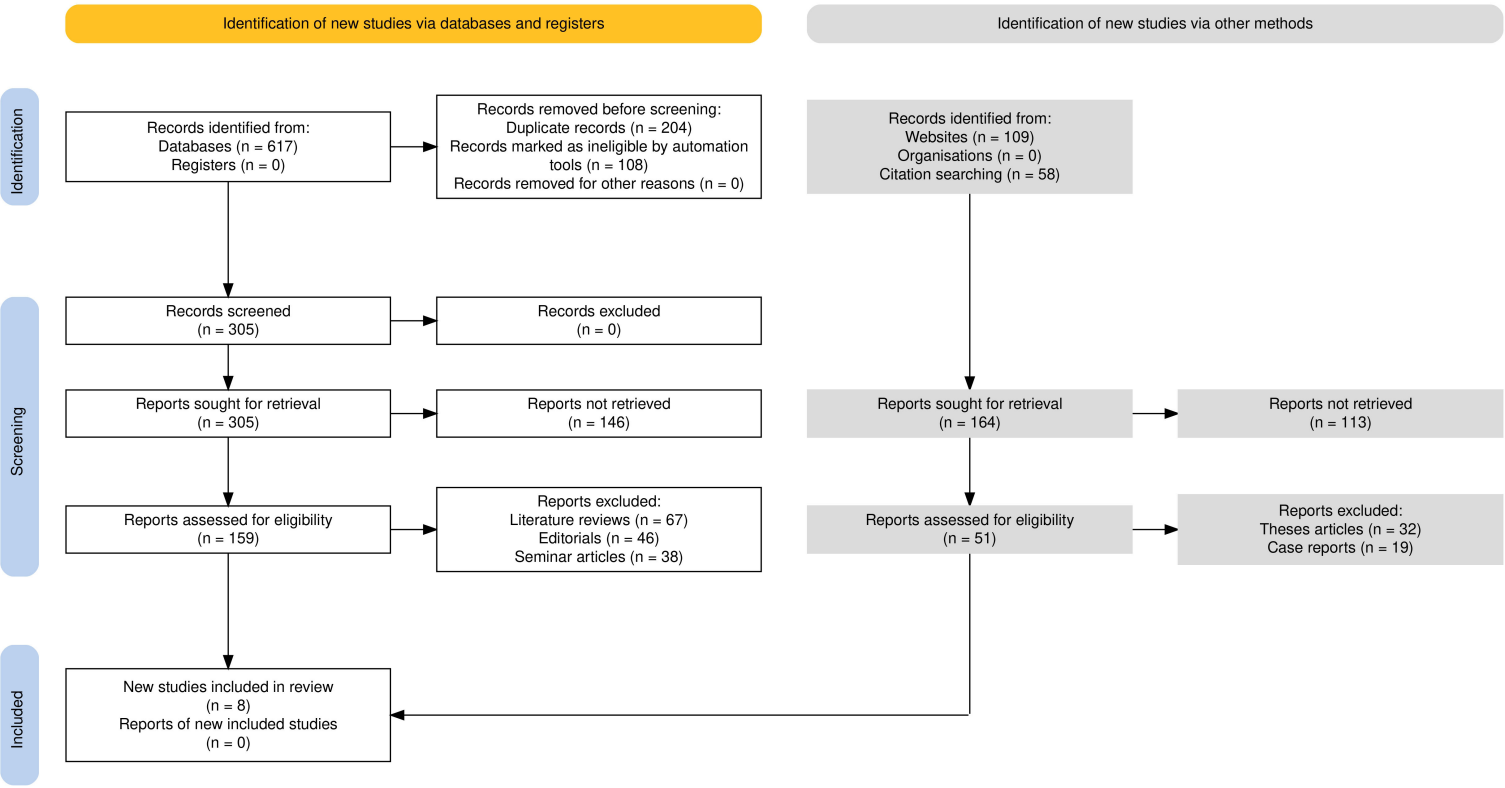
Flowchart representing the PRISMA guideline application in this review.

In this investigation, the study design adhered to the rigorous PRISMA guidelines (11, 12). By adhering to these guidelines, this systematic review ensured transparency, minimized bias, and provided a comprehensive scientific overview of the existing evidence pertaining to the objectives of this study as represented in **Figure 1**. The protocol was registered in the PROSPERO database with the registration number CRD42023428460 being assigned to it.

### PICOS strategy

For the purpose of carrying out this review, a clearly outlined PECOS (Population, Exposure, Comparison, Outcome, Study design) strategy was applied for setting up the research criteria, as mentioned below-

- Population: Cases with prostate cancer (PrCa), diagnosed by tissue analysis or cell cultures.
- Exposure: Expression levels of specific microRNAs (MiRNAs) associated with PrCa progression.
- Comparator: The differences in MiRNA expressions between normal and cancerous tissues or between stages of PrCa.
- Outcomes: Prostate cancer progression markers such as tumor proliferation, metastasis, lymph node dissemination, gene regulation and potential as biomarkers of MiRNA.
- Study design: Experimental studies using quantitative polymerase chain reaction (qPCR) to assess the expression of MiRNA and its impact on PrCa progression.

### Database search strategy

An extensive database search strategy was implemented across seven major online databases. The databases utilized included PubMed/MEDLINE, Embase, Web of Science, Scopus, Cochrane Library, CINAHL, and PsycINFO. A combination of MeSH (Medical Subject Headings) keywords and Boolean operators were employed to ensure a comprehensive search. The MeSH keywords included terms related to PrCa, microRNAs, and cancer progression, such as “prostatic neoplasms,” “microRNAs,” and “disease progression.” Boolean operators (AND, OR) were used to combine these keywords and refine the search results. Additionally, truncation and wildcard symbols were utilized to account for variations in spelling and word endings. **Table 1** displays the search strategy across these databases.

**Table 1:** Online database search using Boolean operators and MeSH keywords.

Database	Search Terms	Boolean Operators
PubMed/MEDLINE	(“prostatic neoplasms”[MeSH Terms] OR “PC”) AND (“microRNAs”[MeSH Terms] OR “MiRNA”)	AND
Embase	(“prostatic neoplasms”/exp OR “PC”) AND (“microRNAs”/exp OR “MiRNA”)	AND
Web of Science	TS=(“prostatic neoplasms” OR “PC”) AND TS=(“microRNAs” OR “MiRNA”)	AND
Scopus	TITLE-ABS-KEY(“prostatic neoplasms” OR “PC”) AND TITLE-ABS-KEY(“microRNAs” OR “MiRNA”)	AND
Cochrane Library	(“prostatic neoplasms” OR “PC”) AND (“microRNAs” OR “MiRNA”)	AND
CINAHL	(MH “prostatic neoplasms” OR “PC”) AND (MH “microRNAs” OR “MiRNA”)	AND
PsycINFO	(“prostatic neoplasms” OR “PC”) AND (“microRNAs” OR “MiRNA”)	AND

### Selection criterion for the review

The inclusion and exclusion criteria for this review were defined to ensure the selection of relevant studies while maintaining a focus on the research question. The inclusion criteria encompassed studies that met the following criteria: (1) focused on human subjects diagnosed with PrCa, (2) examined the expression levels of specific MiRNAs in PrCa tissue/tissue samples, (3) investigated the association between MiRNA expression and PrCa progression or clinical outcomes, (4) included a control or comparison group for comparison purposes, and (5) reported sufficient data or statistical measures to assess the association. Studies that were not published in English, involved animal models, lacked relevant outcomes or data, or were conference abstracts, case reports, reviews, or editorial articles were excluded from the review. The criteria were designed to ensure the inclusion of high-quality studies that provided relevant insights into the association between specific MiRNA expression and PrCa progression while excluding studies that may introduce bias or lack appropriate data for analysis.

### Data extraction and reviewer protocol

The data extraction protocol for this investigation employed multiple reviewers to ensure accuracy and minimize bias. The reviewers were trained in the data extraction process and were provided with a standardized data extraction form. Initially, a pilot test was conducted to ensure consistency and clarity in the extraction process. The reviewers independently extracted relevant data from the selected studies, including study characteristics (e.g., author, year, country), participant characteristics (e.g., sample size, age), methodology (e.g., study design, MiRNA assessment methods), and outcomes (e.g., MiRNA expression levels, PrCa progression measures). Any discrepancies or uncertainties were resolved through discussion and consensus among the reviewers. Additionally, a quality assessment of the included studies was conducted to evaluate the risk of bias and methodological quality. The data extraction process was carefully conducted to ensure the systematic collection of relevant information from each study, and the involvement of multiple reviewers helped to enhance the reliability and validity of the extracted data for further analysis and synthesis in the systematic review.

### Bias assessment of included studies

The bias assessment for this study utilized the Newcastle-Ottawa Scale (NOS). Two independent reviewers assessed the included studies using the NOS criteria (13, 14), which evaluate three main domains: selection of study groups, comparability of groups, and ascertainment of exposure or outcome. Each criterion was scored, and the total scores were used to determine the overall risk of bias for each study. Any disagreements or discrepancies in the assessment were resolved through discussion and consensus between the reviewers. The NOS allowed for a comprehensive evaluation of the included studies’ quality and risk of bias, providing valuable insights into their internal validity and potential sources of bias. This rigorous bias assessment using the NOS ensured that only studies with a high methodological quality and low risk of bias were included in the systematic review, enhancing the reliability and validity of the findings.

### Statistical protocol

The meta-analysis of the studies included in this review was conducted using RevMan 5, a specialized software for meta-analyses in systematic reviews. Data from eligible studies were extracted and synthesized using robust statistical methods. Effect sizes, such as odds ratios (ORs) and risk ratios (RRs), along with their corresponding 95% confidence intervals (CIs), were calculated for the outcomes of interest. The influence of MiRNAs on PrCa progression was assessed based on the total number of tissue samples analyzed for changes associated with MiRNA expression. Statistical heterogeneity among the included studies was evaluated using the I² statistic. In cases of substantial heterogeneity, a random-effects model was employed to pool the data. Sensitivity analyses were performed to assess the impact of individual studies on the overall effect estimates, while subgroup analyses were conducted to explore sources of heterogeneity based on patient characteristics, study design, or other relevant factors. Forest plots were generated to visually represent the meta-analysis results. Overall effect estimates were calculated using the inverse variance method, and statistical significance was evaluated using p-values. The study was registered in PROSPERO with acknowledgement number 428460.

## Results

Initially, a total of 617 records were identified from database searches. No study was retrieved from registers. Before screening, 204 duplicate records were removed and 108 records were excluded as ineligible by automation tools. This left 305 records for screening. All of them were excluded since they did not meet the inclusion criteria based on title and abstract screening. At the screening stage, an attempt was made to retrieve all the 305 records. However, owing to access issues or incomplete publication, retrieval of 146 reports became impossible. The remaining 159 reports were screened for inclusion. At this stage, 67 literature reviews were excluded due to lack of original data; 46 editorials were removed because they did not contain experimental results; and 38 seminar articles were removed from the list as they did not attain methodological standards. In addition, 109 records were found through website searches and 58 through citation searching, for a total of 164 reports retrieved. Of these, 113 could not be obtained because they were unavailable or restricted. The remaining 51 reports were reviewed for inclusion; however, 32 thesis articles were excluded because they were not peer-reviewed articles, and 19 case reports were excluded because they did not present experimental outcomes related to the review. Ultimately, 8 studies (15–22) met the predefined inclusion criteria and were included in the review.

**Figure 2:**
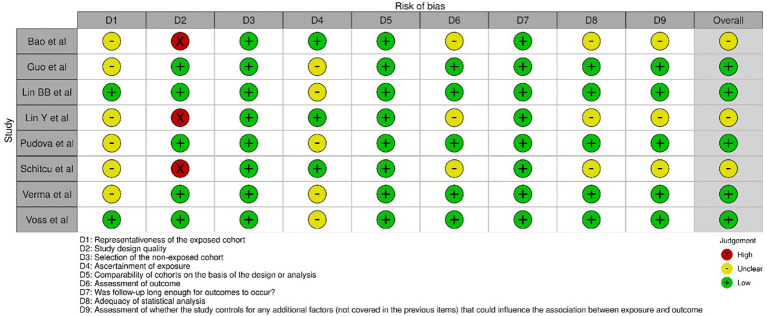
Evaluation of intra-review bias in the included studies.

**Figure 2** presents the assessment of bias of the 8 selected studies (15–22) for the review using the NOS across different domains. The statistical analysis was often rated as low risk of bias, indicating an appropriate and adequate analysis of the data. The assessment of whether the study controls for any additional factors that could influence the association between exposure and outcome was generally rated as unclear, indicating insufficient information on potential confounders. Overall, the studies exhibited some variations in the risk of bias across the different domains as assessed by the NOS. The representativeness of the exposed cohort, assessment of outcome, and control for additional factors were areas where many studies lacked clarity and provided an uncertain risk of bias. However, there were domains, such as selection of the non-exposed cohort, comparability of cohorts, and statistical analysis, where the risk of bias was generally low.

**Table 2** provides a comparison of major assessments conducted across the selected studies. The studies were conducted in different regions and varied in sample size and mean age. The studies conducted in China (15–18) had sample sizes ranging from 60 to 478, and the mean age was unspecified for all of them. Pudova et al’s study (19) in Russia had a smaller sample size of 44 tissue samples, with a mean age of 64 years. Schitcu et al’s study (20) in Romania also had a sample size of 44 participants, with a mean age of 66 ± 4.9 years. Verma et al’s study (21) in the USA utilized 292 MiRNA counts, but the sample mean age was unspecified. Voss et al’s study (22) in Sweden had a sample size of 136 participants, and the mean age was unspecified. These differences in sample size and mean age across the studies reflect the variability in the populations and settings in which the research was conducted.

**Table 2:** Included papers and their characteristics.

Study ID	Year	Region	Sample size (n)	Mean age (in years)
Bao et al. (15)	2017	China	60	Unspecified
Guo et al. (16)	2017	China	70 (tissue samples)	Unspecified
Lin BB et al. (17)	2021	China	478 (tissue samples)	Unspecified
Lin Y et al. (18)	2018	China	142 (tissue samples)	Unspecified
Pudova et al. (19)	2020	Russia	44 (tissue samples)	64
Schitcu et al. (20)	2022	Romania	44	66 ± 4.9
Verma et al. (21)	2019	USA	292 (MiRNA count)	Unspecified
Voss et al. (22)	2020	Sweden	136	Unspecified

**Table 3** presents a comparison of findings and other variables assessed across the selected studies. Regarding the protocol, all studies utilized an experimental approach, indicating a consistent methodology in examining the impact of MiRNAs on PrCa. Multiple MiRNAs were identified across the studies. This indicates a wide exploration of MiRNA involvement in PrCa. The target genes assessed varied across the studies and included FOXO1, FOXO3a, PTEN, TMPRSS2, ERG, TGFβ, PP2A, EGR1, PDCD4, TP53, AGO2, BIRC5, EGFR, DICER1, SSB, NF2, PPARA, and several others. This suggests a comprehensive evaluation of the genes influenced by MiRNAs in PrCa. The gene sequencing method predominantly employed was quantitative polymerase chain reaction (qPCR). This widely used method provided consistent and reliable results across the studies for analyzing MiRNA expression and its impact on target genes. In terms of inference, the studies examined various aspects related to MiRNA involvement in PrCa. These included the effect of MiRNAs on gene expression to prevent or promote PrCa growth, upregulation of MiRNAs in PrCa tissues, potential MiRNAs as biomarkers for early detection of PrCa metastases, correlation between specific MiRNAs and PrCa proliferation and lymph node dissemination, and the influence of MiRNAs on gene regulation in different stages of PrCa. By comparing these variables across the studies, it becomes evident that there is a comprehensive exploration of MiRNA’s role in PrCa. The studies collectively provide insights into the impact of MiRNAs on gene expression, their potential as biomarkers, and their association with PrCa progression. Additionally, some studies specifically highlight the influence of MiRNAs on osteoblast binding and cell adhesion between PrCa cells, indicating their potential impact on PrCa metastasis.

**Table 3:** Results and other different variables assessed pertaining to MiRNA in the included papers.

Study ID	Protocol	MiRNA identified	Target genes assessed	Gene sequencing method	Inference assessed
Bao et al. (15)	Experimental	MiR-96, anti-MiR-96	FOXO1, FOXO3a	qPCR (with DMEM cell culture and 10% FBS)	MiRNAs helped in gene expression that helped prevent PrCa while their suppression promoted the growth of the disease.
Guo et al. (16)	Experimental	MiR-20b	PTEN	qPCR (with cell culture containing 10% FBS, penicillin and streptomycin)	MiRNA analysed in this study were significantly upregulated in PrCa tissues.
Lin BB et al. (17)	Experimental	Unspecified (4 different molecular subtypes were assessed in two different microarray and RNAseq datasets)	TMPRSS2, ERG, TGFβ	qPCR (with cell culture containing 10% FBS, penicillin and streptomycin)	The four types of PrCa subtypes demonstrated substantial potential for diagnosis and treatment guidance based on MiRNA-induced gene regulation.
Lin Y et al. (18)	Experimental	MiR-101-3p, MiR-145-5p, MiR-152, MiR-198, MiR-204-5p	Unspecified	qPCR	The MiRNAs were recognized as potential biomarkers for the early detection of PrCa metastases.
Pudova et al. (19)	Experimental	MiR-7, MiR-10b, MiR-25, MiR-93, MiR-96, MiR-143, MiR-182, MiR-183, MiR-184, MiR-221, MiR-221, MiR-222, MiR-455, MiR-455, MiR-615, MiR-1248, MiR-1271, MiR-181c	PP2A, EGR1, PTEN, PDCD4, FOXO1	qPCR	18 MiRNAs were found to be correlated with PrCa proliferation and lymph node dissemination.
Schitcu et al. (20)	Experimental	MiR-7, MiR-10b, MiR-25, MiR-93, MiR-96, MiR-143, MiR-182, MiR-183, MiR-184, MiR-221, MiR-221, MiR-222, MiR-455, MiR-455, MiR-615, MiR-1248, MiR-1271, MiR-181c	TP53, AGO2, BIRC5, EGFR	qPCR	MiRNAs supported the upregulation and downregulation of genes that significantly influenced PrCa proliferation.
Verma et al. (21)	Experimental	MiR-7, MiR-10b, MiR-25, MiR-93, MiR-96, MiR-143, MiR-182, MiR-183, MiR-184, MiR-221, MiR-221, MiR-222, MiR-455, MiR-455, MiR-615, MiR-1248, MiR-1271, MiR-181c	AGO2, DICER1, SSB, NF2, PPARA	qPCR (with cell culture containing 10% FBS, penicillin and streptomycin)	MiRNAs supported the upregulation and downregulation of genes that significantly influenced PrCa proliferation in its different stages.
Voss et al. (22)	Experimental	MiR-96	ADFP, ACTBL2, SELI, CDH1, HIVEP1, PPM1E, RC3H2, HBEGF, ENC1, CRY1 CCRN4L, KLF6, KIAA1147, GTF2H2	qPCR (with cell culture containing DMEM and 10% FBS)	MiRNA improved osteoblast binding as well as cell adhesion between PrCa cells.

**Figure 3:**
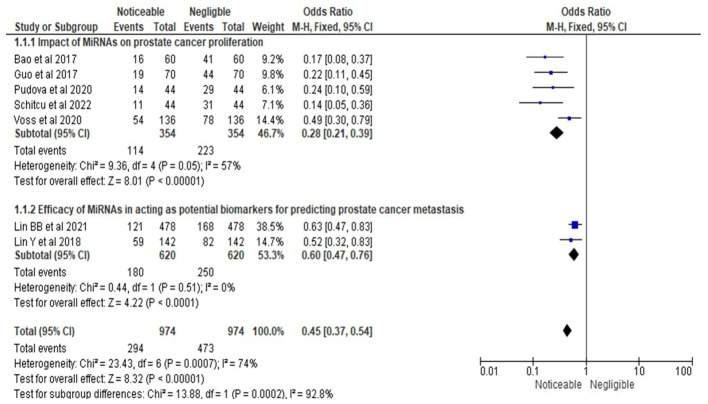
Impact of MiRNAs on PrCa progression in terms of ORs; Efficacy of MiRNAs in acting as potential biomarkers for predicting PrCa metastasis.

As depicted in **Figure 3**, the statistical analysis was conducted for a forest plot demonstrating an OR of 0.28, with a 95% CI ranging from 0.21 to 0.39. This analysis incorporated data from five studies (15, 16, 19, 20, 22), which evaluated the impact of microRNAs (MiRNAs) on prostate cancer (PrCa) proliferation. Proliferation was defined as the measurable increase in tumor growth, assessed using standardized methods such as cell proliferation assays (e.g., MTT assay, BrdU incorporation) or qPCR-based analyses. The results indicated a statistically significant effect of MiRNAs on reducing PrCa proliferation, as evidenced by the OR estimate. Heterogeneity analysis revealed a Chi-square value of 9.36 with 4 degrees of freedom (df) at a significance level of P = 0.05, and an I-squared (I²) statistic of 57%, indicating moderate heterogeneity. This heterogeneity suggested that variations in study design, participant characteristics, or other methodological factors contributed to differences in effect sizes across studies. Furthermore, the overall effect test produced a Z-value of 8.01 with a highly significant P-value of less than 0.00001, confirming a statistically significant overall effect of MiRNAs on PrCa proliferation.

In the second subsection of **Figure 3**, the forest plot revealed an OR of 0.60 with a 95% CI ranging from 0.47 to 0.76, based on two studies (17, 18) investigating the efficacy of MiRNAs as potential biomarkers for predicting PrCa metastasis. Metastasis prediction was defined as the ability of MiRNAs to accurately forecast the spread of cancer cells to secondary locations such as lymph nodes or distant organs, validated through clinical outcomes (e.g., lymph node involvement, progression-free survival) or diagnostic tools such as qPCR or imaging studies. The results demonstrated a statistically significant effect of MiRNAs in predicting metastasis, as indicated by the OR estimate. The heterogeneity analysis reported a Chi-square value of 0.44 with 1 degree of freedom (P = 0.51) and an I² statistic of 0%, indicating no observed heterogeneity. This finding suggested a high degree of consistency in effect sizes across the two studies, likely due to similarities in study design or participant characteristics. The overall effect test produced a Z-value of 4.22 with an extremely significant P-value of less than 0.0001, providing robust evidence for the potential utility of MiRNAs as biomarkers for predicting PrCa metastasis.

**Figure 4:**
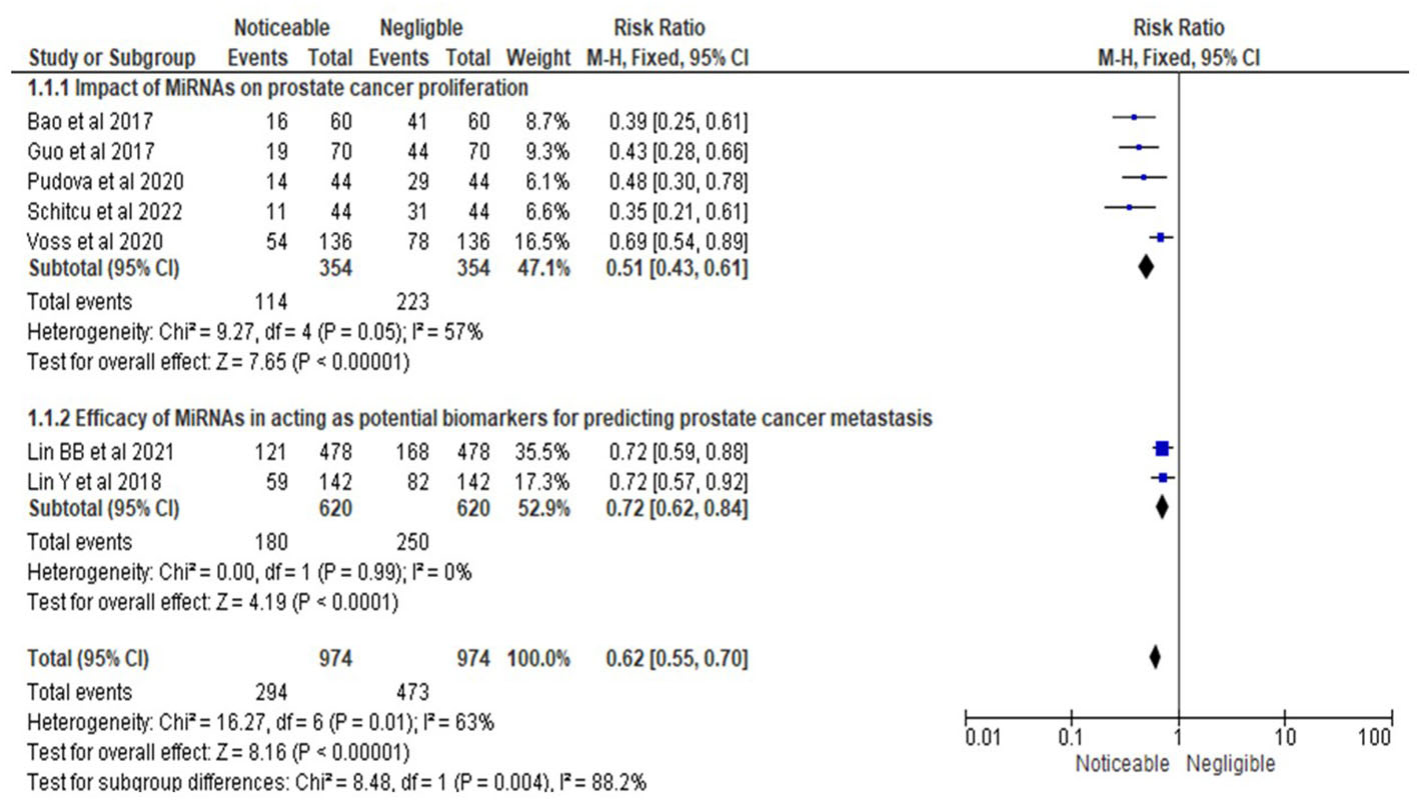
Impact of MiRNAs on PrCa progression in terms of RRs; Efficacy of MiRNAs in acting as potential biomarkers for predicting PrCa metastasis.

Similarly, as shown in **Figure 4**, the statistical analysis of the RR for MiRNAs’ impact on PrCa proliferation yielded an RR of 0.51, with a 95% CI ranging from 0.43 to 0.61. This analysis included data from five studies (15, 16, 19, 20, 22) and confirmed a statistically significant effect of MiRNAs in reducing PrCa proliferation. Proliferation was assessed using standardized definitions and methods, focusing on measurable changes in cell number or tumor growth, as validated through assays or qPCR techniques. Moderate heterogeneity was observed (I² = 57%, Chi-square = 9.27, df = 4, P = 0.05), suggesting some variability in effect sizes attributable to methodological or population-related differences. The overall effect test produced a Z-value of 7.65 with a highly significant P-value of less than 0.00001, supporting the conclusion that MiRNAs significantly influence PrCa proliferation.

In the second subsection of **Figure 4**, the RR for the efficacy of MiRNAs as biomarkers for predicting PrCa metastasis was 0.72, with a 95% CI ranging from 0.62 to 0.84. This analysis incorporated two studies (17, 18), which demonstrated a statistically significant effect of MiRNAs in predicting metastasis. Metastasis prediction was defined and evaluated through standardized criteria, including the ability to predict cancer cell spread using validated diagnostic tools and clinically relevant outcomes. No heterogeneity was detected (I² = 0%, Chi-square = 0.00, df = 1, P = 0.99), indicating consistent findings across the studies. The overall effect test yielded a Z-value of 4.19 with an extremely significant P-value of less than 0.0001, providing strong evidence of MiRNAs’ efficacy as biomarkers for metastasis prediction.

### Sensitivity analysis observations

Despite differences in study settings, all studies employed a qPCR-based experimental approach to evaluate the expression of specific miRNAs and their associations with prostate cancer (PrCa) progression. This methodological consistency minimized the likelihood of bias related to gene expression measurement techniques. The sensitivity analysis was performed by systematically removing each study, one at a time, to observe its impact on the pooled effect sizes for proliferation and metastasis prediction.

1. **Impact on Proliferation Results (OR = 0.28, 95% CI: 0.21–0.39)**
  - The removal of Lin BB et al. (17), which had the largest sample size (n = 478), slightly increased the heterogeneity (I² = 62%) but did not significantly alter the overall effect size. This finding confirmed that the large sample size contributed to the precision of the pooled result but did not disproportionately influence the outcome.
  - Studies with smaller sample sizes, such as Pudova et al. (19) and Schitcu et al. (20), had minimal impact on the effect estimate when excluded, reinforcing the stability of the pooled odds ratio.
  - The heterogeneity remained at a moderate level (I² = 57%) throughout, indicating that the observed variability was due to intrinsic study differences such as sample size, target genes assessed, or population characteristics rather than any single study’s influence.
2. **Impact on Metastasis Prediction Results (OR = 0.60, 95% CI: 0.47–0.76)**
  - The exclusion of each individual study in this analysis, including Lin Y et al. (18) and Lin BB et al. (17), resulted in no significant changes to the pooled odds ratio or heterogeneity.
  - Heterogeneity remained at 0% across all iterations, reflecting a high degree of consistency among the included studies investigating metastasis. This uniformity was likely due to similarities in study design and participant characteristics across these studies.
3. **Impact on Risk Ratios for Proliferation (RR = 0.51, 95% CI: 0.43–0.61)**
  - The analysis for risk ratios showed similar trends, with minimal shifts in the pooled effect size and moderate heterogeneity (I² = 57%). Exclusion of any study, including the larger studies such as Verma et al. (21) and Voss et al. (22), did not undermine the statistical significance of the results.
  - The findings highlighted that the pooled risk ratios remained robust, further validating the association between miRNA expression and reduced prostate cancer proliferation.

## Discussion

The comprehensive exploration of MiRNA involvement in prostate cancer through various experimental studies and the identification of multiple MiRNAs and target genes contribute to our understanding of the molecular mechanisms underlying PrCa development and progression. The studies address various literature gaps by investigating the effects of MiRNAs on gene expression, their potential as biomarkers for early detection of PrCa metastases, their correlation with prostate cancer proliferation and lymph node dissemination, and their influence on gene regulation in different stages of PrCa. The statistical analysis revealed a noticeable effect of MiRNAs on prostate cancer proliferation, as indicated by the OR and RR estimate. Although heterogeneity exists among the studies, the overall effect test confirms a statistically significant association between MiRNAs and PrCa proliferation. Similarly, the efficacy of MiRNAs as potential biomarkers for predicting PrCa metastasis is again demonstrated by the forest plots. The low heterogeneity and highly significant overall effect test provide strong evidence of the potential of MiRNAs as biomarkers for predicting PrCa metastasis. These findings have important implications for future research and clinical practice. The identified MiRNAs and their target genes can serve as potential therapeutic targets or diagnostic biomarkers for PrCa. Further investigation into the specific mechanisms through which MiRNAs regulate gene expression in different stages of PrCa can provide insights into the underlying molecular pathways and potentially lead to the development of targeted therapies. Additionally, the differential efficacy of MiRNAs in predicting PrCa metastasis highlights the need for further research to identify specific MiRNAs with higher accuracy and reliability as biomarkers.

The interplay between miRNAs and key regulators, such as AGO2 (23), involves intricate mechanisms (24). TP53 is involved in regulating miRNA association with AGO2, highlighting a novel pathway for miRNA regulation in carcinogenesis (25, 26). Recent investigations have uncovered the cytoplasmic incorporation of AGO2 and miR-96 (27), shedding light on their potential therapeutic targeting (28). Further exploration has identified miR-542-5p as a notable regulator of AGO2 and EGFR (19, 29, 30). Within the miR-183-96-182 oncogenic cluster, miR-183-5p emerges as a promising diagnostic biomarker for prostate cancer (31–34), while miR-96-5p influences the tumor suppressor PTEN (35). Notably, miR-96 exhibits higher expression levels in prostate cancers with mutant TP53 (36), and its role extends to cell-cell adhesion and androgen signaling regulation (37). Additional investigations implicate miR-20b in prostate cancer progression, with a particular focus on cellular proliferation, epithelial-mesenchymal transition (EMT), and migration (38, 39). Conversely, downregulation of miR-542, especially in TP53 mutated samples, relates to the regulation of EMT, metastasis, and TP53 expression (40). Hypoxic conditions (41) and DNA repair/Notch pathway regulation further influence the expression level of miR-542 (42, 43).

The study has several limitations that should be considered. Firstly, although the protocol of the included studies was consistent in utilizing an experimental approach, there may be variations in the study design and participant characteristics that could contribute to heterogeneity in the findings. Secondly, the assessment of multiple MiRNAs and target genes across the studies provides a comprehensive evaluation, but it may also lead to differences in the specific MiRNAs and genes examined, making it challenging to draw definitive conclusions about the overall role of MiRNAs in PrCa. Additionally, the studies primarily focused on examining MiRNA involvement in PrCa proliferation and its potential as biomarkers, but other important aspects such as MiRNA function in metastasis could be further explored. Furthermore, using ORs and RRs introduced the potential for ORs to overestimate associations when outcomes were common, and RRs being unsuitable for case-control studies. Both measures could have affected by confounding variables if not properly adjusted and provided limited insight into causality. Additionally, heterogeneity across studies could impact their reliability and generalizability. Future research should address these limitations by considering larger sample sizes, standardized study designs, and comprehensive assessments of MiRNA functions in various aspects of PrCa.

## Conclusion

The findings suggest that MiRNAs have a noticeable impact on PrCa proliferation and highlight their potential as biomarkers for predicting PrCa metastasis. However, the presence of heterogeneity among the studies indicates variations in effect sizes that may arise from differences in study design and participant characteristics. Despite this heterogeneity, the statistical analyses confirm a statistically significant overall effect of MiRNAs on PrCa proliferation and their potential as biomarkers for predicting PrCa metastasis. Further research addressing the limitations and exploring MiRNA functions in different aspects of PrCa is warranted to enhance our understanding of MiRNA’s role in this disease.

## Data Availability

The original contributions presented in the study are included in the article/supplementary material. Further inquiries can be directed to the corresponding author.
